# Analysis of factors affecting prognosis of the visual acuity and baseline risk factors for subretinal fibrosis in neovascular age-related macular degeneration patients

**DOI:** 10.3389/fmed.2024.1451726

**Published:** 2024-11-28

**Authors:** Shihan Liu, Minming Zheng, Huixin Sun, Chunxing Pan, Danting Li, Xiyuan Zhou, Zheng Zheng

**Affiliations:** Department of Ophthalmology, The Second Affiliated Hospital of Chongqing Medical University, Chongqing, China

**Keywords:** subretinal fibrosis, neovascular age-related macular degeneration, risk factors, optical coherence tomography, optical coherence tomography angiography

## Abstract

**Background:**

To evaluate factors affecting visual acuity prognosis in patients with neovascular age-related macular degeneration (nAMD) following anti-vascular endothelial growth factor (anti-VEGF) therapy via intravitreal injection and to identify baseline risk factors for subretinal fibrosis (SF).

**Methods:**

A retrospective study of 64 nAMD eyes treated with intravitreal anti-VEGF treatment over 12 months of follow-up was conducted. Demographic and optical coherence tomography characteristics at baseline were recorded to explore the relevant factors affecting visual acuity outcome. Find baseline risk factors for SF development. The primary baseline measures included OCT qualitative and quantitative indicators, and optical coherence tomography angiography (OCTA) quantitative features.

**Results:**

BCVA (logMAR) at 12 months was positively correlated with age (*r* = 0.258, *p* = 0.040), baseline BCVA (*r* = 0.749, *p* < 0.001), central macular thickness (CMT) (*r* = 0.413, *p* < 0.001), subretinal hyperreflective material (SHRM) (*r* = 0.304, *p* = 0.014), intraretinal fluid (IRF) (*r* = 0.423, *p* < 0.001), type 2 macular neovascularization (MNV) (*r* = 0.272, *p* = 0.029), and ellipsoidal zone breaks (*r* = 0.299, *p* = 0.016), and hyperreflective foci (HF) (*r* = 0.264, *p* = 0.035). Eyes with SF had worse baseline BCVA (*p* < 0.001), greater CMT (*p* = 0.009), and a higher prevalence of IRF (*p* = 0.005), type 2 MNV (*p* = 0.001), SHRM (*p* = 0.012), and HF (*p* = 0.028). Logistic binary regression analysis showed that baseline BCVA (logMAR) (OR = 0.02, 95% CI: 0.00–0.45, *p* = 0.013), HF (OR = 0.11, 95% CI: 0.01–0.95, *p* = 0.045), and type 2 MNV (OR = 0.08, 95% CI: 0.01–0.88, *p* = 0.039) were independent risk factors of subretinal fibrosis. As for quantitative OCTA parameters, eyes with subretinal fibrosis had a larger microvascular lesion size (*p* = 0.003), larger vessels area (*p* = 0.002), higher number of vessel junctions (*p* = 0.042) and endpoints (*p* = 0.024), longer total vessel length (*p* = 0.005), and lower vessel length density (*p* = 0.042).

**Conclusion:**

This study enplores baseline OCT and OCTA characteristics associated with subretinal fibrosis in nAMD patients. This information can help predict the occurrence and progression of subretinal fibrosis, potentially leading to more personalized treatment approaches for nAMD patients.

## 1 Introduction

Subretinal fibrosis (SF) is the most common complication of neovascular age-related macular degeneration (nAMD). It causes permanent damage to the retinal pigment epithelium (RPE) and photoreceptors, leading to poor visual acuity in patients with nAMD in the late stages of the disease (1–4). In nAMD, subretinal fibrosis arises from chronic tissue repair attempts. In an inflammatory mediator-rich environment, this repair process involves the recruitment, activation, and proliferation of various cell types, such as immune cells and myofibroblasts. This ultimately leads to excessive deposition and remodeling of the extracellular matrix (ECM) (5). Over time, the neovascular lesion can develop into a fibrovascular complex that progressively forms macular fibrosis (6).

Previous studies have reported a 45% risk of eyes with neovascular age-related macular degeneration (nAMD) developing subretinal fibrosis while receiving anti-vascular endothelial growth factor (anti-VEGF) therapy for 2 years (7). It has also been documented that at the end of the 10-year follow-up period, 62.7% of eyes present signs of fibrosis, with an overall incidence of fibrosis development of 8.9/100 person-years (8). Anti-VEGF therapy is currently the first-line treatment option for nAMD patients. While it can reduce the activity of macular neovascularization (MNV) lesions, it cannot prevent the development of fibrosis and the consequent damage to RPE and photoreceptors (9, 10).

Wu et al. (11) proposed that subretinal fibrosis can serve as a biomarker for predicting incomplete response to anti-VEGF therapy in nAMD. Currently, there is no definitive and effective anti-fibrosis treatment regimen, so early identification of risk factors for subretinal fibrosis can aid in predicting its occurrence and progression, leading to the development of more personalized treatment strategies. Currently, the diagnosis of subretinal fibrosis primarily relies on optical coherence tomography (OCT), color fundus photography, and fluorescein angiography (FA) (7, 12, 13). As a high-resolution imaging technology, optical coherence tomography angiography (OCTA) enables the visualization of the vascular network within the fibrotic scars, as well as changes in the collateral structures of the outer retinal and choroidal vessels (14). Previous studies have tried to identify the risk factors associated with the development of SF, such as type 2 MNV, intraretinal fluid, retinal hemorrhage, and subretinal hyper-reflective material (13, 15–17). However, research on biological markers in OCTA remains scarce.

The present study primarily aimed to investigate baseline characteristics associated with the development of SF. Additionally, we employed AngioTool, a novel software for vascular analysis, to conduct a comprehensive evaluation of the impact of baseline quantitative OCTA features on the development of SF. We also sought to elucidate the factors influencing visual acuity outcomes in patients with nAMD.

## 2 Methods

### 2.1 Study design and participants

This retrospective study was conducted at the Department of Ophthalmology, Second Affiliated Hospital of Chongqing Medical University, between January 2018 and December 2023. A total of 64 participants (64 eyes) were ultimately enrolled, all with follow-up exceeding 12 months. The study adhered to the tenets of the Declaration of Helsinki and received ethical approval from a local institutional review board. Written informed consent was obtained from all patients.

The inclusion criteria for this study were as follows: (1) patients aged 50 years or older diagnosed with nAMD confirmed by both FA and indocyanine green angiography (ICGA); (2) patients who received intravitreal anti-VEGF therapy with a “*pro re nata*” (as needed) treatment regimen (18); and (3) patients with a follow-up period exceeding 12 months. Exclusion criteria included: (1) patients with SF diagnosed at baseline; (2) patients who underwent retinal photocoagulation, photodynamic therapy, or other intraocular surgeries during the follow-up period or previously; (3) patients with diabetic retinopathy, idiopathic choroidal neovascularization, high myopia (greater than −6.00 diopters), or other retinal diseases; and (4) patients with ocular diseases significantly affecting the refractive medium, such as severe glaucoma, cataract, or vitreous hemorrhage. Patients with missing clinical or imaging data were also excluded. In the study, six patients with pre-existing SF were excluded at baseline.

### 2.2 Data collection and instruments

At each visit, all patients underwent a comprehensive ophthalmic examination, including best-corrected visual acuity (BCVA) testing using the international standard logarithmic visual acuity scale (ISLVS), slit-lamp examination, ultra-wide angle fundus photography, spectral-domain optical coherence tomography (SD-OCT), and OCTA. FA and ICGA were performed at baseline presentation and the 12-month follow-up. All diagnoses were made by professional ophthalmologists.

Demographic information, including patients’ age and sex, type of anti-VEGF agent used were collected by reviewing their electronic medical records. BCVA was measured at baseline and each follow-up visit. Baseline imaging studies were analyzed for the following characteristics: MNV type using FA, ICGA, OCT and OCTA; the presence of retinal hemorrhage on fundus photographs; central macular thickness (CMT); and the presence of subretinal fluid (SRF), intraretinal fluid (IRF), subretinal hyperreflective material (SHRM), pigment epithelium detachment (PED), ellipsoid zone loss, abnormal vitreomacular interface, and hyperreflective foci (HF) on OCT. Quantitative microvascular analysis was performed on baseline OCTA images.

### 2.3 Imaging analysis and fibrosis grading

SD-OCT examinations were performed using a CIRRUS™ HD-OCT 5000 machine (Carl Zeiss Meditec, Jena, Germany) with a light source wavelength of 840 nm and a scanning speed of 27,000 A-scans per second. The OCT mode involved a Macular Cube (512 × 128 pixels) centered on the fovea covering a 6 × 6 mm macular region. 12 OCT images with different scanning directions were generated for each OCT examination. All examinations were conducted by the same technician, and image-related parameters were independently evaluated and measured by two investigators. In cases of significant discrepancies, a third senior physician re-evaluated and measured the parameters.

**Figure 1:**
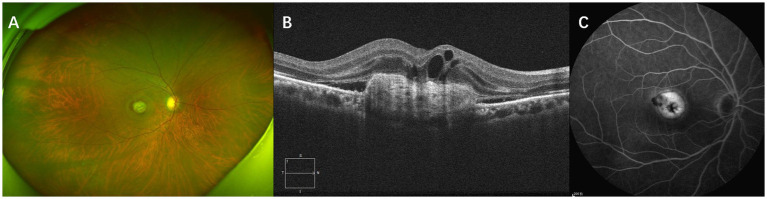
Multimodal imaging of subretinal fibrosis of a 71-year-old male patient suffering from neovascular age-related macular degeneration. The type of MNV lesion is type 2 MNV. IRF, SRF and SHRM were present at baseline. **(A)** Baseline ultrawide-angle fundus photography showing a well-defined, solid, yellow-greenish tissue. **(B)** SD-OCT image showing a dense, homogeneous, well-defined subretinal hyperreflectivity with RPE loss, disruption of the ellipsoid zone (EZ), and the external limiting membrane (ELM). **(C)** An FA image of this patient showing well-defined hyperfluorescence caused by staining of fibrotic scar tissue.

Subretinal fibrosis was identified using a combination of ultrawide-angle fundus photography, SD-OCT, and FA. In ultrawide-angle fundus photos, SF appears as a well-defined, solid, yellow-greenish tissue (12). On SD-OCT, SF manifests as a dense, homogeneous, plaque-like hyperreflective layer beneath the retina with well-defined borders situated between the retinal neurosensory layer and the RPE/Bruch’s membrane complex. In areas with fibrosis, evidence of RPE loss, disruption of the ellipsoid zone (EZ), and the external limiting membrane (ELM) can also be observed (15, 19). FA may show well-defined hyperfluorescence due to staining of the lesion without progressive leakage (20). Figure 1 presents multimodal images of subretinal fibrosis in an 84-year-old woman with nAMD.

Based on SD-OCT, OCTA, FA, and ICGA, neovascularization was classified as type 1 (sub-RPE), type 2 (subretinal), type 3 (retinal angiomatous proliferation, RAP), or polypoid choroidal vasculopathy (PCV) (21).

**Figure 2:**
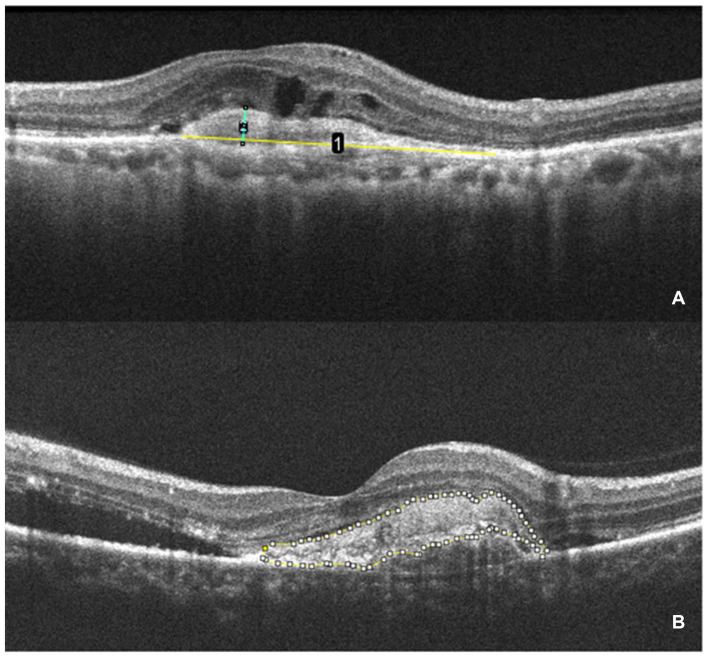
The measurement of SHRM. **(A)** OCT scan showing the maximum width (yellow line 1) and thickness (green line 2) of the SHRM which measured with ImageJ. **(B)** The area of SHRM was also measured with ImageJ. The yellow line traces the area of SHRM, and the software automatically calculates the area value.

CMT is defined as the vertical distance from the internal limiting membrane of the fovea center to Bruch’s membrane. It was measured using ImageJ (version 1.53 g) software. SHRM is defined on OCT as a highly reflective signal located between the retinal neurosensory layer and the retinal pigment epithelium. It typically consists of macular neovascularization components, blood, lipids, exudates, and fibrin (22, 23). Images capturing horizontal and vertical cross-sections through the foveal center of patients were obtained. The maximum width, thickness, and area of SHRM were measured using ImageJ (version 1.53 g) software. These measurements were taken three times to obtain an average value. The final values were determined by averaging the measurements from both the horizontal and vertical cross-sections. Figure 2 presents an example of SHRM quantification.

**Figure 3:**
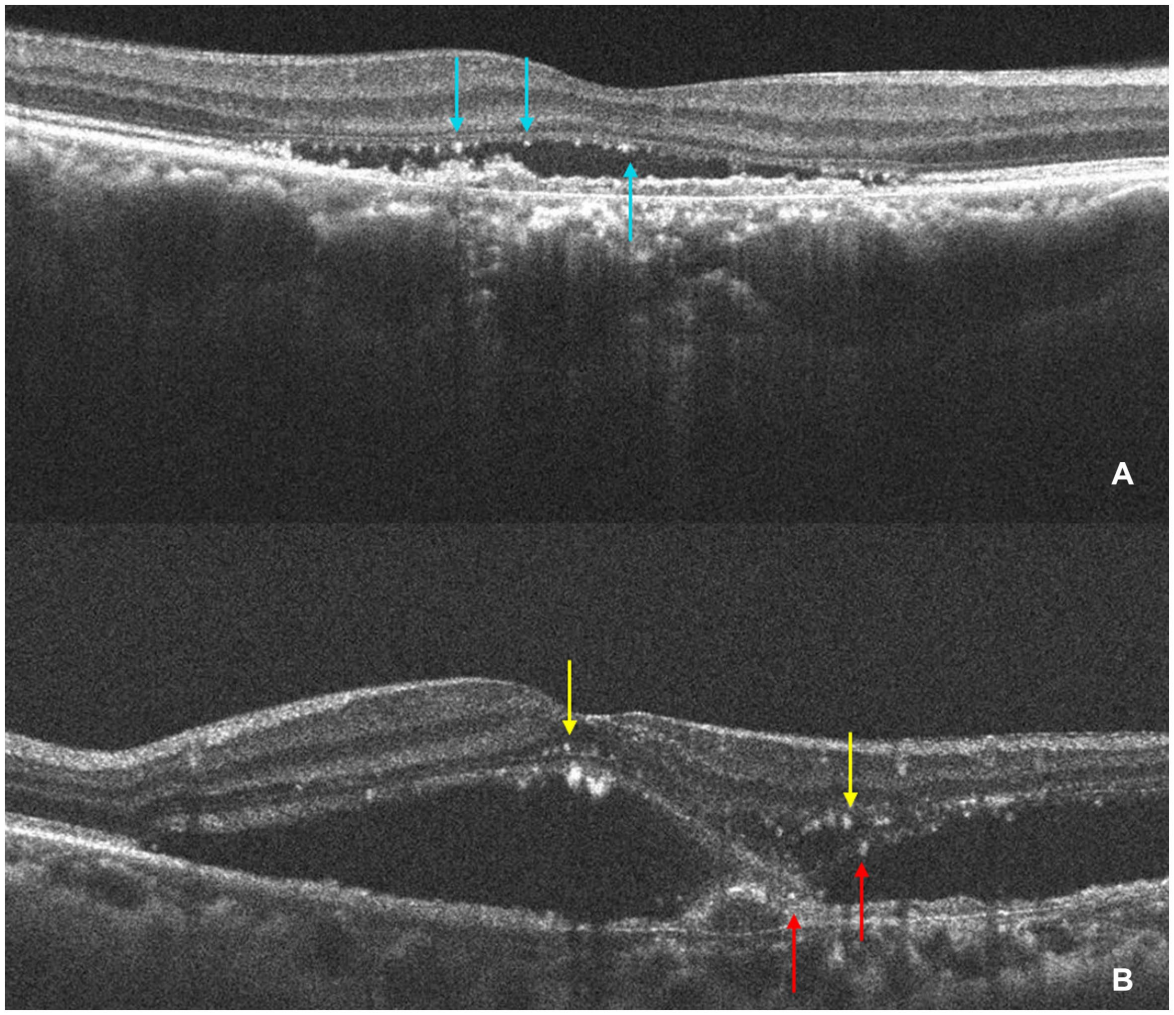
The identification and counting of HF. **(A)** The OCT shows HF (light blue arrows) are concentrated in the subretinal layer and subretinal HF are aggregated at the roof of SRF. **(B)** The OCT shows HF in the inner retina (yellow arrow) and a small amount of subretinal HF (red arrow). The HF was manually counted by the investigators at the same scanning level.

In SD-OCT, hyperreflective foci are defined as scattered, well-circumscribed, dot-shaped lesions with a diameter of less than 50 μm. Usually, they are not fused into a patch and exhibit equal or higher reflectivity compared to the RPE band (24). Investigators manually counted the hyperreflective foci at the same scanning level. The HFs were then categorized according to their positional distribution: inner retina (internal limiting membrane to outer nuclear layer), outer retina (ELM to EZ), and subretina (SRF or MNV to RPE). Figure 3 presents an example of identification and counting of HF.

### 2.4 Quantitative OCTA image analysis

OCTA examinations were performed using a CIRRUS™ HD-OCT 5000 device (Carl Zeiss Meditec, Jena, Germany). The macular region was scanned in the hemodynamic imaging scanning mode. To evaluate the MNV, macular cubes of 6 × 6 mm scan patterns were used. Images with signal intensity below 6, motion artifacts, eyes lacking readily identifiable macular neovascularization (e.g., those with significant pigment epithelium detachment or subretinal hemorrhage in the central fovea), and images with poor foveal center localization were excluded. Ultimately, 42 baseline OCTA images from 42 eyes were included for quantitative analysis of MNV. To generate custom en face images for the MNV analyzes, we manually chose the boundaries of MNV-containing slabs (MNV slab) from the lower edge of the outer nuclear layer to the line of Bruch’s membrane.

The image processing steps were as follows: OCTA images were imported into ImageJ (version 1.53 g) for manual cropping to segment the MNV lesions. Background artifacts were removed using the “Subtract Background” function. Subsequently, the MNV lesions were quantitatively analyzed using AngioTool software (version 0.6a, National Cancer Institute, USA).

AngioTool, a Java-based software for quantitative analysis, offers a user-friendly interface and analysis workflow specifically designed for studying angiogenesis. It identifies vascular structures based on user-defined parameters like vessel diameter and intensity. The software enhances these structures through multiscale Hessian analysis and recursive Gaussian filter smoothing. Finally, it optimizes parameters to skeletonize the vascular network, enabling the calculation of nine parameters. These include lesion area (occupied by the MNV complex), vessels area (containing blood flow within the lesion), percentage of vessels area (vascular area divided by lesion area), number of junctions, junction density (number of junctions per unit lesion area), total and average vessel length, number of endpoints (open vessel ends), and mean E lacunarity (25, 26). These parameters reflect the microvascular characteristics and network inhomogeneity (27). Additionally, we calculated endpoint density (number of endpoints divided by total vessel length). Due to skeletonization with AngioTool, vessel density was expressed as vessel length density (total vessel length divided by vessel area) for better accuracy rather than using the percentage area of vessels.

**Figure 4:**
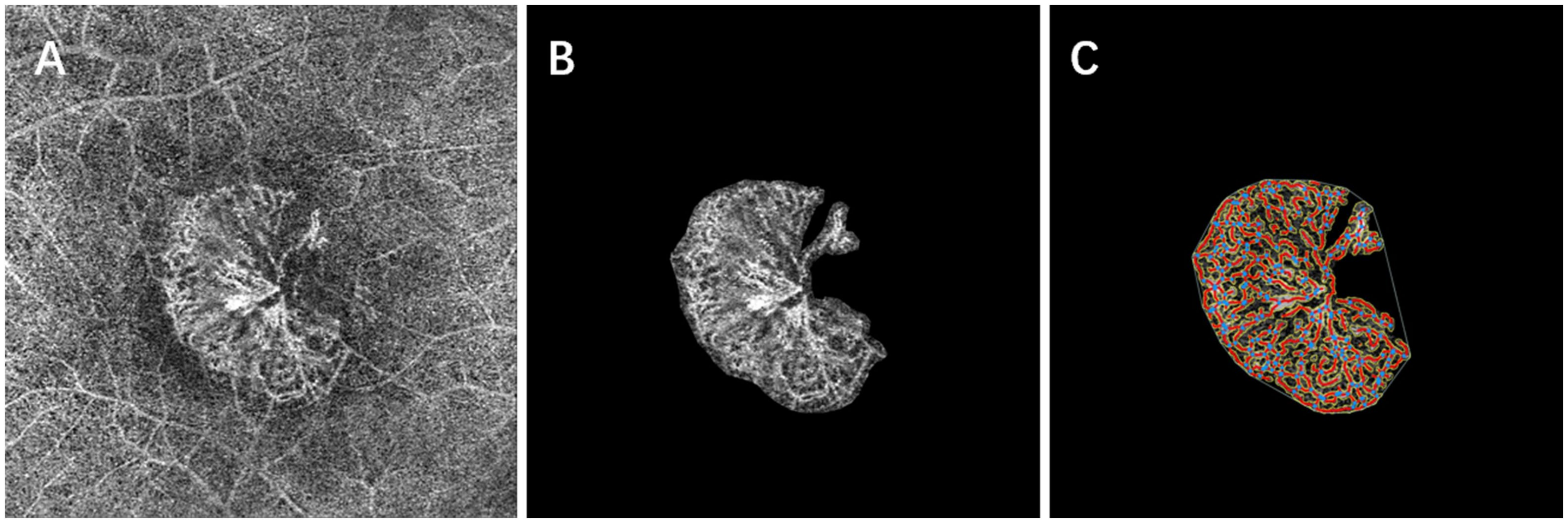
Baseline en face OCT angiography **(A)** shows a subfoveal neovascular net. After careful manual segmentation and background removal of MNV lesions in Image J **(B)**, microvascular complexes were quantitatively analyzed using the AngioTool program, and the results of the analysis **(C)** show the skeletonized vessels (red lines), vessel junctions (blue dots), lesion size (outer white line), and endpoints (open vessel point).

The parameter settings were as follows: low threshold parameters ranged from 15 to 23, the high threshold parameter was set at 255, vessel thickness ranged from 4 to 7, and removal of small particles was set between 0 and 105. Figure 4 details the image processing steps.

### 2.5 Statistical analysis

Statistical analysis was performed using IBM SPSS Statistics 27.0 software. Continuous data with normal distribution were expressed as mean ± SD. Continuous variables that were not normally distributed were described as median (interquartile range, IQR: P25 to P75). Categorical variables were presented as counts (percentage, %). Spearman’s rank correlation analysis was used to assess correlations. For comparisons between groups, independent-sample t-tests were used for normally distributed continuous variables, while the Mann–Whitney U test was used for non-normally distributed continuous variables. Categorical variables were compared using Pearson’s chi-square test or Fisher’s exact test, depending on the sample size. Multivariable binary logistic regression analysis was employed to identify independent risk factors for subretinal fibrosis. No adjustment for multiple testing was performed, as the goals of the study are exploratory rather than confirmatory. A *p*-value of less than 0.05 was statistically significant.

## 3 Results

A total of 64 eyes from 64 patients (34 males, 53.12%) with nAMD were included in the present study. The mean age at treatment initiation was 72.39 ± 9.63 years. Among these eyes, 21 (32.81%) were diagnosed with type 1 MNV, 15 (23.44%) with type 2 MNV, 8 (12.5%) with type 3 MNV, and 20 (31.25%) with PCV. The mean baseline BCVA measured in logMAR units was 0.82 (IQR: 0.40–1.00). All patients were followed for at least 12 months, with a median of 4 injections administered. No serious adverse events, such as cerebrovascular attack or myocardial infarction, were reported in any of the patients.

### 3.1 Visual analysis

Baseline predictors of BCVA at the 12-month follow-up were evaluated using Spearman’s correlation analysis. Significant positive correlations were observed between 12-month BCVA (logMAR) and factors including baseline BCVA (logMAR) (*r* = 0.749, *p* < 0.001), patient age (*r* = 0.258, *p* = 0.040), CMT (*r* = 0.413, *p* < 0.001), SHRM (*r* = 0.304, *p* = 0.014), IRF (*r* = 0.423, *p* < 0.001), type 2 MNV (*r* = 0.272, *p* = 0.029), ellipsoid zone breaks (*r* = 0.299, *p* = 0.016), and hyperreflective foci (*r* = 0.264, *p* = 0.035). Moreover, the 12-month BCVA (logMAR) is not correlated with the other factors included in the analysis (Table 1).

**Table 1:** Analysis of factors correlated to the 12-month BCVA (logMAR) after the first treatment.

Baseline features	Variable value	*r*	*p*
Age	72.39 ± 9.63	0.258	0.040*
Gender, male, *n* (%)	34 (53.12)	0.077	0.547
History of HTN or DM or HPL, *n* (%)	38 (59.38)	−0.094	0.462
Ever smokers, *n* (%)	17 (26.56)	−0.107	0.400
Number of injections	4.00 (3.00,5.00)	0.019	0.880
BCVA, (logMAR)	0.82 (0.40,1.00)	0.749	<0.001*
CMT(μm)	307.79 (204.66,457.83)	0.413	<0.001*
SRF, *n* (%)	31 (48.44)	0.063	0.621
IRF, *n* (%)	17 (26.56)	0.423	<0.001*
SHRM, *n* (%)	39 (60.94)	0.304	0.014*
PED, *n* (%)	28 (43.75)	−0.066	0.604
Type1, *n* (%)	21 (32.81)	−0.239	0.057
Type2, *n* (%)	15 (23.44)	0.272	0.029*
Type3, *n* (%)	8 (12.50)	0.073	0.565
PCV, *n* (%)	20 (31.25)	−0.059	0.645
EZ loss, *n* (%)	40 (62.50)	0.299	0.016*
VMA/VMT, *n* (%)	16 (25.00)	0.017	0.896
HF, *n* (%)	37 (57.81)	0.264	0.035*
Retinal hemorrhage, *n* (%)	13 (20.31)	0.233	0.064

HTN, hypertension; DM, diabetes; HPL, hyperlipidemia; BCVA, best corrected visual acuity; logMAR, logarithm of the minimum angle of resolution; CMT, central macular thickness; IRF, intraretinal fluid; SRF, subretinal fluid; SHRM, subretinal hyperreflective material; PED, pigment epithelium detachment; PCV, polypoidal choroidal vasculopathy; EZ, ellipsoid zone; VMA, vitreomacular adhesion; VMT, vitreomacular traction; HF, hyperreflective foci; *Statistically significant (*p* < 0.05).

**Figure 5:**
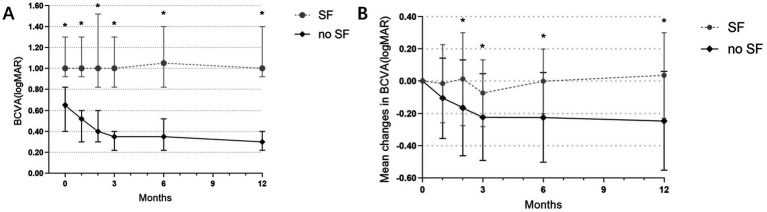
**(A)** Evolution of BCVA (logMAR) at each visit in the SF and non-SF groups before and after anti-VEGF treatment. *The difference in BCVA between the two groups was statistically significant. **(B)** Changes in mean BCVA before and after treatment in both groups. *The difference in the change in mean BCVA (logMAR) between the two groups was statistically significant.

Eyes were categorized based on whether subretinal fibrosis developed during the 12-month follow-up period. The non-fibrotic (non-SF) group comprised 48 eyes, while the subretinal fibrosis (SF) group included 16 eyes. BCVA measured in logMAR units was significantly lower in the non-SF group compared to the SF group at each observation time point from baseline to 12 months (all *p* < 0.001) (Figure 5A).

To account for potential baseline visual acuity differences between the groups, we compared the average changes in BCVA (logMAR) relative to baseline at various follow-up visits (at 1, 2, 3, 6, and 12 months). Significant differences (*p* = 0.041, *p* = 0.048, *p* = 0.004, *p* = 0.002) were observed in the average BCVA (logMAR) changes between the SF group and the non-SF group at 2, 3, 6, and 12 months follow-up visits. The non-SF group exhibited significantly greater improvements in visual acuity compared to the SF group. At 12 months, the non-SF group demonstrated a significant improvement in BCVA, while the SF group even experienced a decrease in BCVA (mean changes in logMAR −0.246 vs. 0.035, *p* = 0.002) (Figure 5B).

### 3.2 Risk factors for fibrosis

No significant differences were found between the fibrosis and non-fibrosis groups in terms of gender (*p* = 0.148), age (*p* = 0.345), smoking history (*p* = 1.000), number of injections in 1 year (*p* = 0.407), type of anti-VEGF drugs used (*p* = 0.655), or other factors. However, baseline BCVA (logMAR) was significantly worse in the non-SF group compared to the SF group (logMAR 0.65 vs. 1.00, IQR: 0.30–0.94 vs. 0.98–1.30, *p* < 0.001). Among the OCT features, statistically significant differences were observed between the groups for IRF (*p* = 0.005), SHRM (*p* = 0.012), HF (*p* = 0.028), and MNV type (distribution of type 2 MNV) (*p* = 0.001). The SF group exhibited a higher prevalence of IRF, SHRM, HF, and type 2 MNV. Additionally, baseline CMT was significantly greater in the SF group compared to the non-SF group (452.25 vs. 286.25 μm, IQR: 331.87–552.92 vs. 192.25–371.00 μm, *p* = 0.009) (Table 2).

**Table 2:** Baseline risk factor analysis for fibrosis.

Baseline features	SF (*n* = 16)	No SF (*n* = 48)	*p*
Age	74.38 ± 10.72	71.73 ± 9.26	0.345
Gender, male, *n* (%)	6 (37.50)	28 (58.33)	0.148
History of HTN or DM or HPL, *n* (%)	7 (43.75)	31 (64.58)	0.142
Ever smokers, *n* (%)	12 (75.00)	35 (72.92)	1.000
Drug			0.655
Conbercept	7 (43.75)	25 (52.08)	
Aflibercept	2 (12.50)	8 (16.67)	
Ranibizumab	7 (43.75)	15 (31.25)	
Number of injections	4.00 (3.00, 4.25)	4.00 (3.00, 5.25)	0.407
BCVA (LogMAR)	1.00 (0.98, 1.30)	0.65 (0.30, 0.94)	<0.001*
CMT (μm)	452.25 (331.87, 552.92)	286.25 (192.25, 371.00)	0.009*
SRF, *n* (%)	8 (50.00)	23 (47.92)	0.885
IRF, *n* (%)	9 (56.25)	8 (16.67)	0.005*
SHRM, *n* (%)	14 (87.50)	25 (52.08)	0.012*
HF, *n* (%)	13 (81.25)	24 (50.00)	0.028*
Lesion type			
Type1, *n* (%)	3 (18.75)	18 (37.50)	0.167
Type2, *n* (%)	9 (56.25)	6 (12.50)	0.001*
Type3, *n* (%)	0 (0.00)	8 (16.67)	0.190
PCV, *n* (%)	4 (25.00)	16 (33.33)	0.533
PED, *n* (%)	4 (25.00)	24 (50.00)	0.081
Ez loss, *n* (%)	13 (81.25)	27 (56.25)	0.074
Retinal hemorrhage, *n* (%)	5 (31.25)	8 (16.67)	0.370

*Statistically significant (*p* < 0.05).

Baseline quantitative indicators of SHRM were compared between the SF and non-SF groups. Fourteen eyes in the SF group and twenty-five eyes in the non-SF group had baseline SHRM. The SF group exhibited a significantly larger SHRM area (*p* = 0.034) and maximum width (*p* = 0.008) compared to the non-SF group (Table 3).

**Table 3:** Comparison of SHRM quantitative indicators between the SF and non-SF groups.

Parameters	SF (*n* = 14)	No SF (*n* = 25)	*p*
SHRM area (μm^2^)	169100.05 (110555.10, 234760.53)	67061.00 (51609.63, 170581.75)	0.034*
SHRM width (μm)	1158.95 (822.71, 1821.65)	566.88 (359.26, 1164.43)	0.008*
SHRM thickness (μm)	199.94 (149.84, 231.26)	189.00 (119.25, 246.79)	0.716

*Statistically significant (*p* < 0.05).

The total number of HF and the number of HF specifically located in the inner retina were significantly higher in the SF group compared to the non-SF group (*p* = 0.044 and *p* = 0.012, respectively). However, the number of HF in the outer or subretinal layers did not differ significantly between the groups (*p* = 0.854) (Table 4).

**Table 4:** Comparative analysis of HF abundance between the fibrosis group and non-fibrosis group.

No. of HF	SF (*n* = 13)	No SF (*n* = 24)	*p*
HF total	4.00 (3.00, 7.00)	2.00 (2.00, 5.00)	0.044*
HF inner	2.00 (0.00, 3.25)	0.00 (0.00, 1.00)	0.012*
HF outer/ subretina	2.50 (0.00, 5.00)	2.00 (0.25, 4.75)	0.854

No. = number; *statistically significant (*p* < 0.05).

In the multifactorial binary logistic regression analysis, which included statistically significant indicators from the univariate analysis, BCVA (logMAR), HF, and type 2 MNV remained statistically significant. These findings suggested that BCVA (logMAR) (OR: 0.02; 95% CI: 0.00–0.45; *p* = 0.013), HF (OR: 0.11; 95% CI: 0.01–0.95; *p* = 0.045), and type 2 MNV (OR: 0.08; 95% CI: 0.01–0.88; *p* = 0.039) were independent risk factors for the development of subretinal fibrosis in nAMD patients following anti-VEGF therapy (Table 5).

**Table 5:** Multivariate binary logistic regression analysis of baseline features associated with subretinal fibrosis.

Parameters	*β*	*p*	OR	95% confidence interval (CI)
BCVA(logMAR)	−3.80	0.013*	0.02	0.00–0.45
IRF	0.09	0.925	1.10	0.15–7.89
SHRM	−0.72	0.484	0.49	0.06–3.67
HF	−2.22	0.045*	0.11	0.01–0.95
Type 2 MNV	−2.48	0.039*	0.08	0.01–0.88

All parameters with univariate *p* <0.05 were analyzed.

### 3.3 Quantitative OCTA analysis

Forty-two baseline OCTA images from 42 eyes were included in the quantitative analysis (32 eyes from the non-SF group and 10 eyes from the SF group). Compared to the non-SF group, the SF group exhibited a significantly larger lesion area (*p* = 0.003) and vessels area (*p* = 0.002). Additionally, the SF group had a greater number of vessel junctions (*p* = 0.042), endpoints (*p* = 0.024), and total vessel length (*p* = 0.005). Interestingly, the vessel length density (*p* = 0.042) was lower in the SF group compared to the non-SF group (Table 6).

**Table 6:** Comparative analysis of OCTA quantitative indicators between the fibrosis group and non-fibrosis group.

Quantitative MNV features	SF (*n* = 10)	No SF (*n* = 32)	*p*
Lesion area (mm^2^)	2.66 (0.85, 3.82)	0.87 (0.24, 1.66)	0.003*
Vessels area (mm^2^)	1.03 (0.34, 1.89)	0.28 (0.10, 0.59)	0.002*
Vessels percentage area (%)	44.55 (34.22, 47.28)	41.42 (31.90, 48.25)	1.000
Number of Junctions (*n*)	103.00 (79.25, 248.50)	62.50 (24.75, 156.30)	0.042*
Junction density (n/mm)	5.43 (4.18, 6.97)	6.67 (5.58, 7.56)	0.084
Total vessel length (mm)	20.24 (12.26, 46.93)	9.58 (3.66, 15.34)	0.005*
Average vessel length (mm)	0.98 (0.66, 3.86)	0.98 (0.66, 3.86)	0.575
Number of endpoints (*n*)	97.00 (63.50,129.50)	54.50 (24.25,84.50)	0.024*
Mean E lacunarity	0.22 (0.16, 0.37)	0.21 (0.14, 0.46)	0.813
Vessel length density (mm/mm^2^)	26.54 (19.56, 35.51)	33.24 (30.96, 38.12)	0.042*
Endpoint density (n/mm)	5.61 (2.26, 6.33)	5.94 (3.91, 7.99)	0.125

*Statistically significant (*p* < 0.05).

## 4 Discussion

Subretinal fibrosis poses a significant threat to visual acuity in patients with nAMD due to its irreversible damage to the photoreceptor and pigment epithelial layers. A recent meta-analysis demonstrated that, despite anti-VEGF treatment, BCVA in eyes with fibrosis was 27 letters worse than in eyes without fibrosis at 12 months (28). Our visual acuity analysis confirms this negative impact. At 12 months, the non-SF group showed a significant improvement in visual acuity from baseline, while the SF group even experienced a decrease. Therefore, investigating baseline predictors of subretinal fibrosis is crucial for both visual acuity outcomes and individualized treatment plans for patients. Herein, we identified several potential risk factors for fibrosis development, including baseline BCVA, CMT, IRF, SHRM, HF, and type 2 MNV. Multivariate binary logistic regression analysis revealed that baseline BCVA, HF, and type 2 MNV were independent risk factors for subretinal fibrosis development.

Previous studies have shown an association between increased HF and RPE atrophy, a precursor to geographic atrophy (29, 30). To our knowledge, this is the first study to identify HF as an independent risk factor for fibrosis development. The SF group exhibited a significantly higher total number of HF and a higher number specifically in the inner retina compared to the non-SF group. Current research suggests that hyperreflective foci may be extravasations caused by blood-retinal barrier damage, represent inflammation-activated microglia, migrating retinal pigment epithelium cells or degenerated photoreceptor cells. Eyes with a higher number of HF may reflect a more severe inflammatory response and greater damage to the RPE or blood-retinal barrier (24, 31). Since fibrosis is more likely in type 2 MNV with disruption of the outer retinal barrier and the inflammatory response is strongly linked to subretinal fibrosis development, we hypothesize that the presence of hyperreflective foci increases the risk of fibrosis.

Consistent with prior studies (13, 15), our findings substantiated that the presence of type 2 MNV at baseline was an independent risk factor for subretinal fibrosis development. This association is likely linked to the disruption of the RPE cell layer. When RPE intercellular contacts are lost, epithelial cells can transdifferentiate into myofibroblasts, ultimately leading to fibrosis. Compared to type 1 MNV, type 2 MNV is more likely to contain damaged and dispersed RPE that undergoes “epithelial-mesenchymal transition.” This process contributes to excessive extracellular matrix deposition and remodeling, potentially explaining the increased risk of fibrosis observed with type 2 MNV (8). Furthermore, our study found that eyes in the fibrosis group had worse baseline BCVA. This finding may be related to the higher prevalence of the more aggressive and vision-threatening type 2 MNV in this group.

SHRM is a known biomarker for predicting visual acuity outcomes in patients with nAMD and has also been identified as a risk factor for fibrosis development (13, 32). Our findings support this notion. SHRM acts as a barrier between the retinal neurosensory layer and the RPE, disrupting metabolism and nutrient exchange. This can impair the overlying photoreceptors through toxic effects, ultimately leading to vision loss. Studies suggest that a well-defined SHRM border on OCT indicates fibrotic tissue or mature neovascular complexes (22). With extended anti-VEGF treatment, the volume of SHRM fluid may decrease relatively, while fibrotic components might increase. The reflectivity of these components tends to rise over time, resulting in sharper boundaries. Casalino et al. (33) proposed that SHRM thickness and width are crucial risk factors for fibrotic scarring in the macula. Alex D et al. (22) observed in their study that a baseline SHRM width exceeding 1,500 μm correlated with a higher incidence of fibrotic scarring. Our study found that the maximum width and area of SHRM in the SF group were significantly greater compared to the non-SF group. We hypothesize that the larger width and area of SHRM indicate more severe damage to photoreceptors and RPE, a longer RPE tear, and a greater number of damaged RPE cells, all of which contribute to a higher risk of progression to fibrosis.

Llorente-Gonzalez et al. (34) reported that subretinal fluid halved the risk of fibrosis development in nAMD, while IRF tripled the risk. Our study similarly identified IRF as a risk factor for fibrosis but found no statistically significant difference in SRF between the groups. The presence of IRF at baseline might suggest more invasive MNV lesions, leading to compromised intraretinal barrier function. Consequently, fluid leakage from the neovascularization may readily enter the retinal neuroepithelial interlayer (35). In this study, type 2 MNV was observed in 58.82% of 17 eyes with baseline IRF and 10.64% of 47 eyes without baseline IRF, with a statistically significant difference (*p* < 0.001). This further suggests that the increased risk of fibrosis associated with IRF may be linked to the presence of a higher proportion of type 2 MNV.

Our study also identified several baseline factors correlated with long-term visual acuity following anti-VEGF therapy, including age, baseline BCVA, CMT, SHRM, IRF, type 2 MNV, ellipsoid zone disruption, and hyperreflective foci. These findings are consistent with previous research (31, 36, 37).

OCTA is a new imaging technology and an extension of OCT technology. It provides depth-resolved visualization of the retinal and choroidal vasculature without the need for dye injection (38). OCT biomarkers focus on the lesion itself, whereas OCTA can be able to evaluate microvascular structures within the lesion in detail. In recent years, some studies have employed OCTA for quantitative assessment of the neovascular network within fibrotic scars. However, research on the correlation between baseline OCTA parameters and subretinal fibrosis development remains limited and faces practical application challenges. A prospective study by Robert et al. (16) found no baseline predictors of fibrosis through quantitative MNV analysis. While they observed a larger baseline lesion area, a greater number of junctions and endpoints, and a longer total vessel length in the SF group, none reached statistical significance. Our study benefitted from a larger sample size compared to Robert et al.’s work. Additionally, we employed AngioTool software for vessel skeletonization, leading to more precise data. This allowed us to find, for the first time, several baseline OCTA microvascular quantitative indices as predictors of fibrosis development: larger MNV lesion area and vessels area, longer total vessel length, higher number of junctions and endpoints, and lower vessel length density. Previous studies have shown that lesion area and vessel area can be used to determine the size of the neovascular network, and vessel junction density and endpoint density can be used to assess the maturity of the neovascular network (39). These findings suggest that larger MNV complexes and more complex microvascular networks at baseline are predictive of fibrosis.

We found a difference between eyes with and eyes without SF in total lesion area and total vessels area. This supports the findings of Bloch et al. that greater neovascularization area is a risk predictor for the development of SF (15). The higher total number of vessel junctions and endpoints and the longer total vessel length in the SF group may be expected because of the larger total lesion area and total vessel area.

In a previous study of MNV morphologic features, the presence of tiny branching vessels and a peripheral anastomotic arcade were suggested to be biomarkers of active lesions (40). Nakano and associates reported higher junction density in type 2 MNV than type 1 MNV using OCTA, which suggests that type 1 MNV vessels are more mature than type 2 MNV vessels (41). Previous studies have also shown that the neovascular network patterns inside a fibrous scar is dominated by “pruned vascular tree,” consisting only of mature trunk vessels, with no thinner capillaries visible (42). But this does not indicate that type 1 MNV, which is more mature than type 2 MNV, is more susceptible to fibrosis. Because anti-VEGF treatment can remodel and mature the vasculature by pruning neovascular buds and promoting vessels with fewer branches, and the microvascular changes involved are influenced by a variety of factors (37). Previous studies have shown vascular flow remodeling induced by recurrent anti-VEGF therapy as well as distinct growth patterns of MNV lesions with treatment (43). To our knowledge, no prior research has elucidated the correlation between baseline maturity of the neovascular network and the development of subretinal fibrosis. Robert et al. suggested that the differences in microvascular features between eyes with and eyes without SF develop over time and may not be present at first presentation (16). Our study found that the fibrosis group had a higher number of both vascular junctions and endpoints compared to the non-fibrosis group. Interestingly, junction density and endpoint density did not differ significantly between the two groups. Therefore, studies on the correlation between baseline maturity of the neovascular network and the development of SF need to include larger sample sizes for analysis, and the importance of these metrics in predicting fibrosis development warrants further investigation.

Our study also revealed that eyes in the SF group had a lower baseline vessel length density. Histologically, neovascular lesions are intricate structures composed of various elements, including blood vessels, macrophages, myofibroblasts, and fibroblasts (44). The process of fibrotic scar formation, known as the vascular-fibrotic transition, involves a decrease in vascular cellularity alongside an increase in fibroblasts. This suggests that fibrosis may develop as neovascularization subsides. Consequently, eyes with lower vascular density might be at a higher risk for vascular-to-fibrotic conversion.

This study has several limitations. First, it is a retrospective design with a relatively small sample size and an unequal distribution of subjects between the two groups. Second, during OCTA image inclusion, we excluded images with motion artifacts or undetectable MNV lesions (partially obscured by hemorrhage or exudate). This process may have introduced some selection bias. Strengths of this study include the use of multimodal imaging for reliable SF detection and comprehensive quantitative analysis of neovascularization using the novel AngioTool software.

## 5 Conclusion

Overall, by utilizing novel multimodal imaging and analysis software, our study achieved two key findings in patients with nAMD treated with anti-VEGF therapy. First, we investigated factors influencing visual acuity outcomes. Second, we enplored risk factors for subretinal fibrosis development. Patients with low baseline BCVA, high CMT, IRF, HF, SHRM, and type 2 MNV exhibited an increased risk of fibrosis. These patients require close follow-up and potentially intensified anti-VEGF therapy. Furthermore, our study found that eyes with nAMD that developed fibrosis had larger and wider SHRM areas and a higher baseline HF count. Additionally, we achieved a first by identifying quantitative OCTA microvascular indicators of subretinal fibrosis development using AngioTool, a semi-automated analysis software.

## Data Availability

The original contributions presented in the study are included in the article/Supplementary material, further inquiries can be directed to the corresponding author.

## References

[1] Wong TY Chakravarthy U Klein R Mitchell P Zlateva G Buggage R. The natural history and prognosis of neovascular age-related macular degeneration: a systematic review of the literature and meta-analysis. Ophthalmology. (2008) 115:116–126.e1. doi: 10.1016/j.ophtha.2007.03.008, PMID: 17675159

[2] Okeagu CU Agrón E Vitale S Domalpally A Chew EY Keenan TDL. Principal cause of poor visual acuity after Neovascular age-related macular degeneration: age-related eye disease study 2 report number 23. Ophthalmol. Retina. (2021) 5:23–31. doi: 10.1016/j.oret.2020.09.025, PMID: 33045457 PMC7796863

[3] Gillies M Arnold J Bhandari S Essex RW Young S Squirrell D. Ten-year treatment outcomes of Neovascular age-related macular degeneration from two regions. Am J Ophthalmol. (2020) 210:116–24. doi: 10.1016/j.ajo.2019.10.007, PMID: 31606444

[4] Cheung CMG Grewal DS Teo KYC Gan A Mohla A Chakravarthy U. The evolution of fibrosis and atrophy and their relationship with visual outcomes in Asian persons with Neovascular age-related macular degeneration. Ophthalmol Retina. (2019) 3:1045–55. doi: 10.1016/j.oret.2019.06.002, PMID: 31444144

[5] Wynn TA. Common and unique mechanisms regulate fibrosis in various fibroproliferative diseases. J Clin Invest. (2007) 117:524–9. doi: 10.1172/JCI31487, PMID: 17332879 PMC1804380

[6] Roberts PK Zotter S Montuoro A Pircher M Baumann B Ritter M. Identification and quantification of the Angiofibrotic switch in Neovascular AMD. Invest Ophthalmol Vis Sci. (2019) 60:304–11. doi: 10.1167/iovs.18-25189, PMID: 30657855

[7] Daniel E Toth CA Grunwald JE Jaffe GJ Martin DF Fine SL. Risk of scar in the comparison of age-related macular degeneration treatments trials. Ophthalmology. (2014) 121:656–66. doi: 10.1016/j.ophtha.2013.10.019, PMID: 24314839 PMC3943618

[8] Romano F Cozzi E Airaldi M Nassisi M Viola F Aretti A. Ten-year incidence of fibrosis and risk factors for its development in Neovascular age-related macular degeneration. Am J Ophthalmol. (2023) 252:170–81. doi: 10.1016/j.ajo.2023.03.033 37030492

[9] Chandra S Arpa C Menon D Khalid H Hamilton R Nicholson L. Ten-year outcomes of antivascular endothelial growth factor therapy in neovascular age-related macular degeneration. Eye (Lond). (2020) 34:1888–96. doi: 10.1038/s41433-020-0764-9, PMID: 31980748 PMC7608465

[10] Toth LA Stevenson M Chakravarthy U. Anti-vascular endothelial growth factor therapy for neovascular age-related macular degeneration: outcomes in eyes with poor initial vision. Retina (Philadelphia, Pa). (2015) 35:1957–63. doi: 10.1097/IAE.0000000000000583 25946692

[11] Wu J Zhang J. Neovascular remodeling and subretinal fibrosis as biomarkers for predicting incomplete response to anti-VEGF therapy in Neovascular age-related macular degeneration. Front Biosci. (2022) 27:135. doi: 10.31083/j.fbl2704135 35468694

[12] Bachmeier I Armendariz BG Yu S Jäger RJ Ebneter A Glittenberg C. Fibrosis in neovascular age-related macular degeneration: a review of definitions based on clinical imaging. Surv Ophthalmol. (2023) 68:835–48. doi: 10.1016/j.survophthal.2023.03.004 37023894

[13] Daniel E Pan W Ying GS Kim BJ Grunwald JE Ferris FL 3rd. Development and course of scars in the comparison of age-related macular degeneration treatments trials. Ophthalmology. (2018) 125:1037–46. doi: 10.1016/j.ophtha.2018.01.004, PMID: 29454660 PMC6015772

[14] Ahmed M Syrine BM Nadia BA Anis M Karim Z Mohamed G. Optical coherence tomography angiography features of macular neovascularization in wet age-related macular degeneration: a cross-sectional study. Ann Med Surg. (2012) 70:102826. doi: 10.1016/j.amsu.2021.102826 PMC8435926 34540215

[15] Bloch SB Lund-Andersen H Sander B Larsen M. Subfoveal fibrosis in eyes with neovascular age-related macular degeneration treated with intravitreal ranibizumab. Am J Ophthalmol. (2013) 156:116–24.e1. doi: 10.1016/j.ajo.2013.02.012 23664150

[16] Roberts PK Schranz M Motschi A Desissaire S Hacker V Pircher M. Baseline predictors for subretinal fibrosis in neovascular age-related macular degeneration. Sci Rep. (2022) 12:88. doi: 10.1038/s41598-021-03716-8, PMID: 34996934 PMC8741927

[17] Sánchez-Monroy J Nguyen V Puzo M Calvo P Arruabarrena C Monaco P. Subretinal fluid may protect against macular atrophy in neovascular age-related macular degeneration: 5 years of follow-up from fight retinal blindness registry. Acta Ophthalmol. (2023) 101:457–64. doi: 10.1111/aos.15309, PMID: 36536538

[18] Kanadani T Rabelo N Takahashi D Magalhães L Farah M. Comparison of antiangiogenic agents (ranibizumab, aflibercept, bevacizumab and ziv-aflibercept) in the therapeutic response to the exudative form of age-related macular degeneration according to the treat-and-extend protocol-true head-to-head study. Int J Retina Vitreous. (2024) 10:13. doi: 10.1186/s40942-024-00537-5 38308362 PMC10836031

[19] Kherani S Scott AW Wenick AS Zimmer-Galler I Brady CJ Sodhi A. Shortest distance from fovea to subfoveal hemorrhage border is important in patients with Neovascular age-related macular degeneration. Am J Ophthalmol. (2018) 189:86–95. doi: 10.1016/j.ajo.2018.02.015, PMID: 29499174 PMC10020831

[20] Evans RN Reeves BC Maguire MG Martin DF Muldrew A Peto T. Associations of variation in retinal thickness with visual acuity and anatomic outcomes in eyes with Neovascular age-related macular degeneration lesions treated with anti-vascular endothelial growth factor agents. JAMA ophthalmol. (2020) 138:1043–51. doi: 10.1001/jamaophthalmol.2020.3001, PMID: 32816002 PMC7441468

[21] Spaide RF Jaffe GJ Sarraf D Freund KB Sadda SR Staurenghi G. Consensus nomenclature for reporting Neovascular age-related macular degeneration data: consensus on Neovascular age-related macular degeneration nomenclature study group. Ophthalmology. (2020) 127:616–36. doi: 10.1016/j.ophtha.2019.11.004 31864668 PMC11559632

[22] Alex D Giridhar A Gopalakrishnan M Indurkhya S Madan S. Subretinal hyperreflective material morphology in neovascular age-related macular degeneration: a case control study. Indian J Ophthalmol. (2021) 69:1862–6. doi: 10.4103/ijo.IJO_3156_20, PMID: 34146045 PMC8374782

[23] Iovino C Ramtohul P Au A Romero-Morales V Sadda S Freund KB. Vitelliform maculopathy: diverse etiologies originating from one common pathway. Surv Ophthalmol. (2023) 68:361–79. doi: 10.1016/j.survophthal.2023.01.009, PMID: 36720370

[24] Lee H Ji B Chung H Kim HC. Correlation between optical coherence tomographic hyperreflective foci and visual outcomes after anti-VEGF treatment in neovascular age-related macular degeneration and Polypoidal choroidal vasculopathy. Retina (Philadelphia, Pa). (2016) 36:465–75. doi: 10.1097/IAE.0000000000000645 26076214

[25] Zudaire E Gambardella L Kurcz C Vermeren S. A computational tool for quantitative analysis of vascular networks. PloS one. (2011) 6:e27385. doi: 10.1371/journal.pone.0027385, PMID: 22110636 PMC3217985

[26] Choi M Kim SW Yun C Oh J. OCT angiography features of neovascularization as predictive factors for frequent recurrence in age-related macular degeneration. Am J Ophthalmol. (2020) 213:109–19. doi: 10.1016/j.ajo.2020.01.012, PMID: 31954711

[27] Schranz M Told R Hacker V Reiter GS Reumueller A Vogl WD. Correlation of vascular and fluid-related parameters in neovascular age-related macular degeneration using deep learning. Acta Ophthalmol. (2023) 101:e95–e105. doi: 10.1111/aos.15219 35912717 PMC10087766

[28] Cheong KX Cheung CMG Teo KYC. Review of fibrosis in Neovascular age-related macular degeneration. Am J Ophthalmol. (2023) 246:192–222. doi: 10.1016/j.ajo.2022.09.008, PMID: 36162537

[29] Leuschen JN Schuman SG Winter KP McCall MN Wong WT Chew EY. Spectral-domain optical coherence tomography characteristics of intermediate age-related macular degeneration. Ophthalmology. (2013) 120:140–50. doi: 10.1016/j.ophtha.2012.07.004 22968145 PMC3536919

[30] Christenbury JG Folgar FA O'Connell RV Chiu SJ Farsiu S Toth CA. Progression of intermediate age-related macular degeneration with proliferation and inner retinal migration of hyperreflective foci. Ophthalmology. (2013) 120:1038–45. doi: 10.1016/j.ophtha.2012.10.018 23352193 PMC3640702

[31] Fragiotta S Abdolrahimzadeh S Dolz-Marco R Sakurada Y Gal-Or O Scuderi G. Significance of Hyperreflective foci as an optical coherence tomography biomarker in retinal diseases: characterization and clinical implications. J Ophthalmol. (2021) 2021:1–10. doi: 10.1155/2021/6096017 PMC8709761 34956669

[32] Finn AP Pistilli M Tai V Daniel E Ying GS Maguire MG. Localized optical coherence tomography precursors of macular atrophy and fibrotic scar in the comparison of age-related macular degeneration treatments trials. Am J Ophthalmol. (2021) 223:338–47. doi: 10.1016/j.ajo.2020.11.002, PMID: 33221285 PMC7979472

[33] Casalino G Stevenson MR Bandello F Chakravarthy U. Tomographic biomarkers predicting progression to fibrosis in treated Neovascular age-related macular degeneration: a multimodal imaging study. Ophthalmol Retina. (2018) 2:451–61. doi: 10.1016/j.oret.2017.08.019, PMID: 31047325

[34] Llorente-González S Hernandez M González-Zamora J Bilbao-Malavé V Fernández-Robredo P Saenz-de-Viteri M. The role of retinal fluid location in atrophy and fibrosis evolution of patients with neovascular age-related macular degeneration long-term treated in real world. Acta Ophthalmol. (2022) 100:e521–31. doi: 10.1111/aos.14905, PMID: 34085771

[35] Călugăru D Călugăru M. Inner nuclear layer cystoid spaces are a poor prognostic factor in typical age-related macular degeneration and polypoidal choroidal vasculopathy. Graefes Arch Clin Exp Ophthalmol. (2018) 256:627–9. doi: 10.1007/s00417-017-3829-0, PMID: 29075861

[36] Kumar JB Stinnett S Han JIL Jaffe GJ. Correlation of SUBRETINAL HYPERREFLECTIVE material morphology and visual acuity in NEOVASCULAR age-related macular degeneration. Retina (Philadelphia, Pa). (2020) 40:845–56. doi: 10.1097/IAE.0000000000002552 31305505

[37] Ying GS Maguire MG Pan W Grunwald JE Daniel E Jaffe GJ. Baseline predictors for five-year visual acuity outcomes in the comparison of AMD treatment trials. Ophthalmol Retina. (2018) 2:525–30. doi: 10.1016/j.oret.2017.10.003, PMID: 29938247 PMC6009839

[38] Spaide RF Klancnik JM Jr Cooney MJ. Retinal vascular layers imaged by fluorescein angiography and optical coherence tomography angiography. JAMA Ophthalmol. (2015) 133:45–50. doi: 10.1001/jamaophthalmol.2014.3616 25317632

[39] Takeuchi J Kataoka K Ito Y Takayama K Yasuma T Kaneko H. Optical coherence tomography angiography to quantify choroidal neovascularization in response to Aflibercept. Ophthalmologica. (2018) 240:90–8. doi: 10.1159/000487611, PMID: 29739007

[40] Coscas F Lupidi M Boulet JF Sellam A Cabral D Serra R. Optical coherence tomography angiography in exudative age-related macular degeneration: a predictive model for treatment decisions. Br J Ophthalmol. (2019) 103:1342–6. doi: 10.1136/bjophthalmol-2018-313065 30467129 PMC6709766

[41] Nakano Y Kataoka K Takeuchi J Fujita A Kaneko H Shimizu H. Vascular maturity of type 1 and type 2 choroidal neovascularization evaluated by optical coherence tomography angiography. PLoS One. (2019) 14:e0216304. doi: 10.1371/journal.pone.0216304 31034505 PMC6488195

[42] Miere A Semoun O Cohen SY El Ameen A Srour M Jung C. Optical coherence tomography angiography features of SUBRETINAL fibrosis in age-related macular degeneration. Retina. (2015) 35:2275–84. doi: 10.1097/IAE.0000000000000819 26457397

[43] Xu D Dávila JP Rahimi M Rebhun CB Alibhai AY. Long-term progression of type 1 neovascularization in age-related macular degeneration using optical coherence tomography angiography. Am J Ophthalmol. 187:10–20. doi: 10.1016/j.ajo.2017.12.005 29269100

[44] Querques L Parravano M Borrelli E Chiaravalloti A Tedeschi M Sacconi R. Anatomical and functional changes in neovascular AMD in remission: comparison of fibrocellular and fibrovascular phenotypes. Br J Ophthalmol. (2020) 104:47–52. doi: 10.1136/bjophthalmol-2018-313685, PMID: 31000509

