# Supplementary material for: Analysis of factors affecting prognosis of the visual acuity and baseline risk factors for subretinal fibrosis in neovascular age-related macular degeneration patients
Source: Front Med (Lausanne). 2024 Nov 28;11:1451726. doi: 10.3389/fmed.2024.1451726 (PMC11634580; doi:10.3389/fmed.2024.1451726)
Supplement: Supplementary file 1 [file Table_1.DOCX]

Supplementary Material

# Supplementary Data

We have attempted multivariate analysis of OCTA quantitative data, and the results suggest that none of the OCTA quantitative parameters reached statistically significant. Second, it is our opinion that the sample size for quantitative analysis of OCTA was small and multivariate statistical analyses may be biased., so we did not present the results of this part of the analysis. We considered placing this part of the result in the supplementary material. Thanks again for your reminder.

Table: Multivariate binary logistic regression analysis of baseline OCTA quantitative indicators associated with subretinal fibrosis.

| **Parameters** | **β** | **P** | **OR** | **95% confidence interval (CI)** | |
| --- | --- | --- | --- | --- | --- |
| Lesion area (mm^2^) | -0.38 | 0.772 | 0.68 | | 0.05 ~ 9.14 |
| Vessels area(mm^2^) | 2.11 | 0.771 | 8.23 | | 0.00 ~ 11.87 |
| Number of Junctions (n) | 0.02 | 0.610 | 1.02 | | 0.94 ~ 1.11 |
| Total vessel length (mm) | -0.24 | 0.675 | 0.78 | | 0.25 ~ 2.44 |
| Number of endpoints (n) | -0.01 | 0.631 | 0.99 | | 0.95 ~ 1.03 |
| Vessel length density (mm/mm^2^) | 0.06 | 0.656 | 1.06 | | 0.82 ~ 1.36 |
